# *Yersinia enterocolitica*: Prevalence, virulence, and antimicrobial resistance from retail and processed meat in Egypt

**DOI:** 10.14202/vetworld.2019.1078-1084

**Published:** 2019-07-21

**Authors:** Gamal Younis, Mona Mady, Amal Awad

**Affiliations:** Department of Bacteriology, Mycology and Immunology, Faculty of Veterinary Medicine, Mansoura University, Mansoura, 35516, Egypt

**Keywords:** antimicrobial susceptibility, biofilm formation, virulence genes, *Yersinia enterocolitica*

## Abstract

**Background and Aim:**

The objectives of this study were to investigate the prevalence of *Yersinia enterocolitica* in retail chicken meat, ground and processed beef meat, determine their virulence-associated genes, antimicrobial susceptibility pattern, molecular detection of extended-spectrum β-lactamases, and their capability of biofilm formation *in vitro*.

**Materials and Methods:**

A total of 210 samples (120 retail chicken meat, 30 ground beef, 30 beef burger, and 30 sausage samples) were collected from different retail chicken outlets and markets located at Mansoura city between December 2016 and April 2017. Meat samples were examined bacteriologically for the existence of *Y. enterocolitica*; bacterial colonies that displayed positive biochemical properties were subjected to polymerase chain reaction targeting 16 rRNA gene. *Y. enterocolitica* isolates were tested for their susceptibility to six antimicrobial agents using disk diffusion method. Uniplex PCR was used for screening *Y. enterocolitica* isolates for the presence of two virulence chromosome-associated genes (*ail* and *yst*), and β-lactamases (*bla*_TEM_ and *bla*_SHV_). The capability of *Y. enterocolitica* to form biofilms was detected by tube method.

**Results:**

Thirty *Y. enterocolitica* isolates (14.29%) were recovered including 19 (15.83%) isolates from chicken meat, 3 (10%) from ground beef, 5 (16.67%) from beef burger, and 3 (10%) from sausage samples. Regarding *ail* gene, it was detected in 6.67% (2/30), while *yst* gene detected in 20% (6/30) *Y. enterocolitica* isolates. About 80%, 70%, 63.33%, and 50% of *Y. enterocolitica* isolates were sensitive to ciprofloxacin, gentamicin, cefotaxime, and streptomycin, respectively, while 83.33% of *Y. enterocolitica* isolates were resistant to both ampicillin and cephalothin. Interestingly, 21 (70%) isolates had the capability of biofilms formation *in vitro*. Among the multidrug-resistant (MDR) strains, a significant difference (p<0.05) was found between MDR and biofilm formation. However, biofilm formation was correlated with the resistance of the isolates to β-lactam antimicrobials and the presence of β-lactam-resistant genes.

**Conclusion:**

The presence of *Y. enterocolitica* in chicken meat, ground and processed beef meat represents a significant health risk for meat consumers, which reflects the contamination of slaughterhouses and processing operations, therefore, strict hygienic measures should be applied to minimize carcasses contamination.

## Introduction

Foodborne diseases are prevalent and growing public health concern worldwide [1]. In Egypt, there is currently a lack of information on the occurrence of foodborne diseases including Yersiniosis. Yersiniosis is a gastrointestinal infection caused by *Yersinia enterocolitica* which is considered the most prevalent gastrointestinal infection after campylobacteriosis and salmonellosis in the industrial countries [2]. *Y. enterocolitica* is a psychotropic waterborne and foodborne enteropathogen that has the capability to grow at refrigeration temperatures [3]. Contaminated meat, chicken, milk, and cheese with *Y. enterocolitica* cause a significant health risk for consumers, especially in young children and infants [4,5].

*Y. enterocolitica* has been isolated from different sources including animals, food, and environment. Pigs are considered the main reservoir for human pathogenic strains of *Y. enterocolitica*, so pigs and pig products are the main sources for human infection [5]. However, poultry meat has been also implicated in *Y. enterocolitica* infections [6] as birds are commonly subclinical carriers of the organism [7].

The most common clinical symptoms of *Y. enterocolitica* infections are enteritis, enterocolitis, acute diarrhea, fever, abdominal pain, mesenteric lymphadenitis, and pseudoappendicitis [8]. It has strong ability for extraintestinal spreading under defined host conditions causing extraintestinal manifestations, including wide range of clinical symptoms [9].

*Y. enterocolitica* strains have the ability to cause the disease due to the presence of different virulence factors located in the chromosome or carried on a 70 kb virulence plasmid (pYV), which is only detected in virulent strains [10]. Two chromosomal virulent-associated genes *(ail* and *yst*) were included in our analysis. The *ail* gene (adhesion invasion locus) encodes a surface factor that enhances epithelial cell penetration, is involved in adhesion and invasion, and is found only among pathogenic strains [11]. The *yst* gene encodes enterotoxin is frequently detected in diarrheagenic biotype 1A strains [12].

Antimicrobial treatment is not necessary for treating *Y. enterocolitica* in immunocompetent hosts because most gastrointestinal infections are self-limiting. However, systemic, extraintestinal, and invasive infections in immunocompromised patients with increased risk for developing bacteremia or septicemia require special attention and antibiotic therapy since the mortality rate in these cases can be as high as 50% [10]. Ciprofloxacin, ceftriaxone, and cefotaxime have been used successfully for complicated infection treatments such as liver abscess, endocarditis, and septicemia [13].

Bacterial biofilm is a community of microorganisms surrounded by a self-produced matrix consisting of an extracellular polymeric substance of polysaccharides, lipids, proteins, and nucleic acids [14]. Biofilm formation protects bacterial cells from antibacterial agents, phagocytes, bacteriophages, and antibodies. In food industry, biofilms increase the resistance of bacteria to many physical and chemical factors which represent a food safety concern. Accordingly, the process of biofilm formation represents a method of protection for microbial growth allows bacterial cells to survive and grow in several adverse environments. In addition, the formation of biofilms may result in chronic infection due to the resistant of pathogens to both host immunity and antibiotic treatment [15].

Therefore, this study was designed (1) to investigate the prevalence rate of *Y. enterocolitica* in chickens meat, ground and processed beef meat samples collected from Mansoura, Egypt, with the determination of its associated chromosomal virulence genes (*ail* and *yst*); (2) to determine the antimicrobial susceptibility patterns, β-lactam resistance genes, and biofilm formation; and (3) to ascertain if there is a relationship between biofilm formation and multidrug-resistant (MDR) isolates.

## Materials and Methods

### Ethical approval

There was no need for ethical approval in this study because no live animals were involved. Meat samples were collected from retail outlets and various supermarkets.

### Samples collection

A total of 210 samples were examined in this study, including 120 retail chicken meat samples, 30 ground beef samples, and 60 processed meat samples (30 beef burger and 30 sausage). Meat samples were chosen randomly from retail outlets and various supermarkets in Mansoura city, Egypt, from December 2016 to April 2017. The collected samples (approximately 50 g each) were kept separately in a sterile plastic bag, labeled, and delivered to the laboratory for bacteriological examinations in an ice container.

### Isolation and identification of *Y. enterocolitica* isolates

About 25 g of each sample was homogenized in 225 ml of peptone, sorbitol, and bile salts broth (PSB; Difco, Heidelberg, Germany) and homogenized in a Stomacher for 2 min at high speed. Homogenized samples were incubated at 25°C for 72 h. Then, a loopful of the enriched samples was streaked into Cefsulodin-Irgasan-Novobiocin agar medium plates (Oxoid) and incubated for 24 h at 30°C under aerobic conditions. Colonies with typical red bull’s eye-like colonies (small, with a red center and translucent rim) were purified on the surface of tryptic soy agar plates (Oxoid) and then stored at 4°C for further examination. Preliminary identification of *Y. enterocolitica* isolates depended on the colony morphological criteria by Gram’s stain and biochemically subjected to the following biochemical tests: catalase, oxidase, urease production, Simmons citrate, triple sugar iron, and sugar fermentation [16]. Furthermore, a 16S rRNA-based polymerase chain reaction (PCR) technique was used for *Y. enterocolitica* confirmation using the primers and the conditions listed in Table-1 [17-20].

**Table 1:** Oligonucleotide primers sequences used in this study.

Target Gene	Primer	Sequence	Annealing	Amplicons size	References
*Y.enterocolitica* 16S rRNA	*Y1*	AATACCGCATAACGTCTTCG	60	330 bp	[17]
	*Y2*	CTTCTTCTGCGAGTAACGTC			
*ail*	*ail-F*	TAATGTGTACGCTGCGAG	55	351 bp	[18]
	*ail-R*	GACGTCTTACTTGCACTG			
*yst*	*Pr2a*	AATGCTGTCTTCATTTGGAGC	55	145 bp	
	*Pr2c*	ATCCCAATCACTACTGACTTC			
*bla*_TEM_	*TEM-F*	ATTCTTGAAGACGAAAGGGC	60	1150	[19]
	*TEM-R*	ACGCTCAGTGGAACGAAAAC			
*bla*_SHV_	*SHV-F*	CACTCAAGGATGTATTGTG	50	885	[20]
	*SHV-R*	TTAGCGTTGCCAGTGCTCG			

*Y.enterocolitica*=*Yersinia enterocolitica*

### Molecular detection of β-lactam resistance genes and virulence-associated genes

The cell lysates from *Y. enterocolitica* isolates were obtained by boiling [7] to be used as DNA templates. Confirmed *Y. enterocolitica* were screened for the presence of two virulence chromosome-associated genes, including adhesion invasion locus gene (*ail*) encoded a surface factor that enhanced epithelial cell penetration and the heat-stable enterotoxin encoded *yst* using the primers and PCR conditions listed in Table-1 [17-20] according to the referred authors. Furthermore, *Y. enterocolitica* isolates were also screened for the presence of β-lactam resistance genes including *bla*_TEM_ and *bla*_SHV_. PCRs were performed with Applied Biosystem, 2720 Thermal Cycler (USA) in a total volume of 25 µL consisted of 12.5 µL of 2× PCR master mix (Promega, Madison, USA), 1 µL of each primer (Metabion, Germany), 4.5 µL PCR-grade water, and 6 µL DNA template. The amplicons were separated in agarose gel 1.5% (Lonza Rockland, ME, USA) in TBE buffer stained with ethidium bromide. Gels were photographed by Gel Doc (Cleaver Scientific Ltd., USA).

### Antimicrobial susceptibility testing

Antimicrobial susceptibility of *Y. enterocolitica* isolates was determined against six different antimicrobials belonged to different antimicrobial classes using Kirby–Bauer disk diffusion method on Mueller–Hinton agar (Oxoid) plates following the Clinical and Laboratory Standards Institute guidelines [21]. The following antimicrobial disks (Oxoid) used were cefotaxime (30 µg), gentamicin (10 µg), streptomycin (10 µg), ciprofloxacin (5 µg), ampicillin (10 µg), and cephalothin (30 µg).

### Biofilm formation

*Y. enterocolitica* isolates were examined for its capability of biofilms production using tube method. In brief, a loopful of each isolate has been inoculated separately in 5 ml trypticase soy broth (TSB; Becton Dickinson, Sparks, USA). The inoculated tubes were incubated at 28°C for 24 h, and then, 1 ml from the incubated broth was transferred into another sterilized 4 ml TSB and incubated at 28°C for 24 h. Uninoculated tube of TSB was used as a control. After incubation, the previously inoculated broth was discarded carefully, and the tubes were stained with 1% crystal violet for 15 min; excess stain was discarded and washed with deionized water. The stained tubes were left to dry in an inverted position and photographed to determine biofilm formation [22].

### Statistical analysis

The variation in the prevalence of virulence genes, beta-lactam-resistant genes, and biofilm formation was calculated by Chi-square test. A statistically significant difference did exist if p<0.05. Pearson’s correlation was used to study the correlation between all studied phenotypic and genotypic features of *Y. enterocolitica* isolates using the R program Ver. 324 (Package: corrplot). For creating the correlation matrix, the numbers “1” and “0” were assigned for the presence and absence of the corresponding genes, respectively.

## Results and Discussion

*Y. enterocolitica* is one of the leading foodborne infections, which also could become a significant health risk for consumers. However, it is not considered among the most frequent food-related pathogens such as *Salmonella* and *Campylobacter*; it is of great concern in terms of food safety as it can survive and grow at refrigeration temperature [23]. In this study, a total of 210 meat samples were examined for the presence of *Y. enterocolitica* using the conventional cultural methods and molecular characterization mentioned. All recovered *Y. enterocolitica* isolates were genetically verified as *Y. enterocolitica* strains by molecular detection of 16S rRNA gene. Among the total examined samples, 30 isolates (14.29%) were identified to be *Y. enterocolitica* (Table-2). The prevalence of *Y. enterocolitica* in chicken meat was 15.83% (19/120). In agreement with our findings, Shabana *et al*. [24] could isolate *Y. enterocolitica* in a prevalence rate of 17.5% from raw chicken meat in Egypt while Momtaz *et al*. [25] found that 18.33% (132/720) chicken meat samples were positive for *Y. enterocolitica* in Iran. Comparing to the obtained results, Anju *et al*. [6] could not identify *Y. enterocolitica* from chicken meat while Sirghani *et al*. [26] recorded a higher percentage (30%) from chicken meat in Iran.

**Table 2:** Isolation rate of *Y. enterocolitica* from the examined meats samples.

Types of samples	Number of examined samples	Number of positive samples	Percentage of positive samples
Chicken meat	120	19	15.83
Ground beef	30	3	10
Beef burger	30	5	16.67
Sausage	30	3	10
Total	210	30	14.29

*Y. enterocolitica*=*Yersinia enterocolitica*

The prevalence rate of *Y. enterocolitica* recovered from ground beef samples was 10% (3/30) which was lower than that detected by Karib *et al*. [27], who found that 15% (3/20) of the ground beef samples were contaminated with *Y. enterocolitica*. The prevalence rate of *Y. enterocolitica* in beef burger samples was 16.67% (5/30), which was higher than that obtained by Nortjé *et al*. [28], while the prevalence rate of *Y. enterocolitica* from sausage samples was 10% (3/30) which was higher than that reported by Nortjé *et al*. [28]. Interestingly, a higher prevalence (16.67%) of *Y. enterocolitica* contamination was recorded from beef burger.

*Y. enterocolitica* that contains virulence factor only has the capability to induce disease [29]. Virulence genes in *Y*. *enterocolitica* located either on chromosome or on a plasmid named pYV. The most significant virulence genes located on chromosome include ail, inv, and yst. Detection of ail gene is a key factor to distinguish pathogenic and non-pathogenic *Y*. *enterocolitica* [30]. In this study, *Y. enterocolitica* isolates were examined for the presence of two chromosomal genes because plasmid pYV is easily lost depending on various factors [31]. The *ail* gene is a type of protein responsible for adhesion and invasion [11], while *yst* is an enterotoxin, commonly found in diarrheagenic biotype 1A strains [12]. The obtained results from this study reveal the presence of *ail* in 6.67% (2/30) of the tested isolates, while *yst* was carried by six strains (20%). The obtained results from the present study were in accordance with Peng *et al*. [32] and lower than that described by Sacchini *et al*. [33], who could detect *ail* and *yst* from all tested *Y. enterocolitica* isolates, while Bhagat and Virdi [34] could not identify both genes in their study. The pathogenicity of *Y. enterocolitica* associated closely with ail gene, biotypes, and serotypes [35]. The diversity in the pathogenicity of *Y. enterocolitica* isolates may be depended on the geographical area of isolation. By testing the correlation between the presence of the two virulence genes used, we found a strong positive correlation between them (*yst* and *ail*: 0.56).

Antimicrobial resistance among *Y. enterocolitica* isolates was tested against ampicillin (AMP), cephalothin (CF), ciprofloxacin (CIP), gentamicin (GN), cefotaxime (CTX), and streptomycin (S). Regarding β-lactam resistance, 83.33% (25/30) of the examined isolates showed resistance to both ampicillin and cephalothin. The tested strains revealed intermediate resistant against streptomycin (50%; 15/30) and a lower resistance was revealed by cefotaxime (36.67%; 11/30) followed by gentamicin (30%; 9/30) and ciprofloxacin (20%; 6/30) as shown in Table-3. Sixteen resistance patterns were detected; the most common pattern was S, CF, AMP represented by eight strains followed by CF and AMP displayed by five strains. MDR to ≥3 of the antimicrobial classes tested was detected in 7 (23.33%) of *Y. enterocolitica* isolates; among them, the most common profile was CTX, S, CF, and AMP.

**Table 3:** Number and percentage of *Y. enterocolitica* antimicrobial pattern isolated from chicken and minced meat.

Antimicrobial agent	Code	Sensitive		Resist	
	
		n	%	n	%
Ciprofloxacin	CIP	24	80	6	20
Gentamicin	GN	21	70	9	30
Cefotaxime	CTX	19	63.33	11	36.67
Streptomycin	S	15	50	15	50
Cephalothin	CF	5	16.66	25	83.33
Ampicillin	AMP	5	16.66	25	83.33

CF=Cephalothin, AMP=Ampicillin, S=Streptomycin, CTX=Cefotaxime, CIP=Ciprofloxacin, GN=Gentamicin, *Y. enterocolitica=Yersinia enterocolitica*

Antimicrobial resistance possesses by Gram-negative bacteria including *Y. enterocolitica* which represents a significant public health problem worldwide. In this study, the antimicrobial resistance of *Y. enterocolitica* isolates was higher in comparison with other studies; it was noticed that *Y. enterocolitica* showed higher rate of ampicillin and cephalothin resistance (83.33% each) which is in agreement with the previous studies [1,33,36,37]; the high percentage of resistance to β-lactam antimicrobial may be attributed to the ability of *Y. enterocolitica* to produce β-lactamases, which contribute to ampicillin and cephalothin resistance [38]. MDR was detected in 23.33% (7/30) of *Y. enterocolitica* isolates, but none of the examined isolates was resistance to all of the tested antimicrobial agents. However, the isolates show high susceptibility to ciprofloxacin, gentamicin, cefotaxime, and streptomycin. The antimicrobial resistance pattern of the isolated *Y. enterocolitica* strains is described in Table-4. By testing the correlation between phenotypic and genotypic antimicrobial susceptibility, we found a positive correlation between the presence of *bla*_TEM_ gene with the phenotypic resistance to ampicillin (R=0.5) and cephalothin (R=0.3), as well as, *bla*_SHV_ correlated positively with the resistance to ampicillin (R=0.3) and cephalothin (R=0.2). In addition, there is positive correlation between both genes (*bla*_TEM_ and *bla*_SHV_: 0.49).

**Table 4:** The distribution of antimicrobial resistance profiles among *Y. enterocolitica* isolates.

Antimicrobial resistance profile	Number of antibiotics	Number of isolates
CIP, CTX, GN, CF, AMP	5	1
CTX, GN, S, CF, AMP	5	1
CTX, S, CF, AMP	4	2
S, AMP, GN, CIP	4	1
CIP, CTX, GN, S	4	1
GN, S, CF, AMP	4	1
CTX, CF, AMP	3	2
GN, CF, AMP	3	2
CIP, CF, AMP	3	1
S, CF, AMP	3	8
GN, S, CF	3	1
CTX, AMP	2	1
CF, AMP	2	5
CTX, CF	2	1
CTX	1	1
CIP	1	1

CF=Cephalothin, AMP=Ampicillin, CTX=Cefotaxime, *Y. enterocolitica*=*Yersinia enterocolitica*

**Figure-1:**
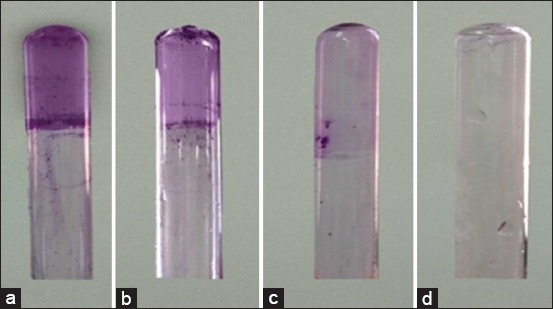
Detection of the degree of biofilm production using tube test. (a) Strong biofilm producer, (b) Moderate biofilm producer, (c) Weak biofilm producer, (d) Non-biofilm producer.

The presence of biofilms could contribute to *Y. enterocolitica* pathogenicity; in this study, 21 (70%) isolates had the capability to form biofilms. Eleven isolates were strong, six isolates were moderate, four isolates were weak biofilm producer (Figure-1), while 30% (9/30) were detected as non-biofilm producer. As previously mentioned, MDR was detected in 7 strains (23.33%) of *Y. enterocolitica* isolates. By testing the relationship between MDR and the capability of biofilm production, we found that there is no relation between MDR and biofilm formation capability with significant differences between the MDR and non-MDR isolates (p<0.05). However, biofilms formation was correlated with the resistance to specific types of antibiotics such of β-lactams including ampicillin (0.25) and cefotaxime (0.25) and it is weakly correlated with the presence of β-lactam-resistant genes (*bla*_SHV_: 0.04). In addition, biofilm formations were correlated with the presence of virulence-associated genes (*yst*: 0.23 and *ail*: 0.13) (Table-5).

**Table 5:** Correlation matrix showing the positive and negative correlation between phenotypic and genotypic features in *Y. enterocolitica* isolates.

Examined characters	*ail*	*yst*	CIP	*bla*_CTX_	GN	S	CF	AMP	*bla*_TEM_	*bla*_SHV_	*Biofilm production*
*ail*	1										
*yst*	0.557086	1									
CIP	−0.09285	−0.16667	1								
*bla*_CTX_	0.244051	0.207514	0.138343	1							
GN	−0.12157	0.024246	0.581914	0.105661	1						
S	−0.1857	−0.33333	−4.6E-18	−0.20751	0.072739	1					
CF	0.083045	0.149071	−0.44721	−0.21654	−0.09759	0.089443	1				
AMP	0.083045	0.149071	−0.22361	−0.21654	−0.09759	0.089443	0.52	1			
*bla*_TEM_	0.162386	−0.15696	−0.06727	−0.17216	−0.16147	0.36336	0.150414	0.511408	1		
*bla*_SHV_	0.212351	0.156957	−0.1009	−0.10702	0.014679	−0.06727	0.21058	0.391077	0.493213	1	
Biofilm formation	0.131306	0.235702	−0.17678	−0.04891	−0.30861	−0.14142	0.252982	0.252982	−0.19026	0.047565	1

The degree of correlation (R) ranges from 0 to 1. 0 indicates the lowest correlation and 1 indicates the highest correlation. The correlation matrix was created using the R program Ver. 324. (Package: Corrplot). CF=Cephalothin, AMP=Ampicillin, *Y. enterocolitica*=*Yersinia enterocolitica*

## Conclusion

The results from this study reveal the contamination of chicken meat, ground and processed beef meat by *Y. enterocolitica* and it’s high resistant to beta-lactams antimicrobials which is considered a potential source of human infection. As a result, strict hygienic measures should be applied to minimize *Y. enterocolitica* contamination in poultry and beef meat.

## Authors’ Contributions

GY designed the study and revised the manuscript. MM and AA collected meat samples, conducted all experiments and wrote the manuscript. All authors read and approved the final manuscript for publication.
